# Supplementary material for: Knowledge and attitudes of medical students towards the health impact of climate change: A study from Jordan
Source: PLoS One. 2025 May 28;20(5):e0324943. doi: 10.1371/journal.pone.0324943 (PMC12118995; doi:10.1371/journal.pone.0324943)
Supplement: Attachment — Submitted filename: Responses to reviewers.docx [file pone.0324943.s003.docx]

**Dear Editor of PLOS-ONE**

We are thankful to the reviewers for their thoughtful and detailed review of our manuscript (ONE-D-24-44537) entitled “Knowledge and attitudes of medical students towards the health impact of climate change: a study from Jordan”. All the recommended changes and edits have been incorporated in the revised version of our manuscript. We greatly appreciate the time and effort invested to produce this detailed review. Our point-by-point response is provided below.

Comment

1. When submitting your revision, we need you to address these additional requirements.

Please ensure that your manuscript meets PLOS ONE's style requirements, including those for file naming. The PLOS ONE style templates can be found at
[https://journals.plos.org/plosone/s/file?id=wjVg/PLOSOne_formatting_sample_main_body.pdf](https://track.editorialmanager.com/CL0/https:%2F%2Fjournals.plos.org%2Fplosone%2Fs%2Ffile%3Fid=wjVg%2FPLOSOne_formatting_sample_main_body.pdf/1/010f019373f31d57-2db1aa2a-c666-47b3-98c6-b6949112ebc8-000000/j6gSi2LE5jWkBE5edmmvBdOibdgQIrHmdHv1zGZgZFo=187) and
[https://journals.plos.org/plosone/s/file?id=ba62/PLOSOne_formatting_sample_title_authors_affiliations.pdf](https://track.editorialmanager.com/CL0/https:%2F%2Fjournals.plos.org%2Fplosone%2Fs%2Ffile%3Fid=ba62%2FPLOSOne_formatting_sample_title_authors_affiliations.pdf/1/010f019373f31d57-2db1aa2a-c666-47b3-98c6-b6949112ebc8-000000/ZZf6byecvRJLDy8Ub9bY9ieKyE0Krv5TEIeuvxToi2U=187)

Response

The manuscript was checked to meet PLOS ONE's style requirements

2. Thank you for stating the following financial disclosure:
[The study was supported by fund from Jordan University of Science and Technology (fund number: 210-2024).].

Please state what role the funders took in the study. If the funders had no role, please state: "The funders had no role in study design, data collection and analysis, decision to publish, or preparation of the manuscript."

If this statement is not correct you must amend it as needed.

Please include this amended Role of Funder statement in your cover letter; we will change the online submission form on your behalf.

Response

We have revised the Financial Disclosure statement as indicated. The revised statement is:

The revised statement is “The study was supported by funds from Jordan University of Science and Technology (fund number: 210-2024) and the GeoHealth Hub for Climate Change and Health in the Middle East and North Africa (Fogarty NIH awards number 5U2RTW012228 and U01TW012237). The funders had no role in study design, data collection and analysis, decision to publish, or preparation of the manuscript.”

Changes to the Financial Disclosure statement were added to the cover letter.

3. Thank you for stating the following in the Acknowledgments Section of your manuscript: [Authors thank Mr. Ahmed Kofahi, Mr. Ali Hisham, and Mr. Ali Alomar for their efforts in the recruitment of participants. Authors would like to thank the GeoHealth Hub for Climate Change and Health in the Middle East and North Africa (#5U2RTW012228 and #U01TW012237) for its support.]

We note that you have provided funding information that is not currently declared in your Funding Statement. However, funding information should not appear in the Acknowledgments section or other areas of your manuscript. We will only publish funding information present in the Funding Statement section of the online submission form.

Please remove any funding-related text from the manuscript and let us know how you would like to update your Funding Statement. Currently, your Funding Statement reads as follows: "The study was supported by fund from Jordan University of Science and Technology (fund number: 210-2024)."

Please include your amended statements within your cover letter; we will change the online submission form on your behalf.

Response

The Acknowledgement statement was revised as suggested. In addition, the financial statement was revised as shown in our response to point 2.

4. In the online submission form, you indicated that your data is available only on request from a third party. Please note that your Data Availability Statement is currently missing the contact details for the third party, such as an email address or a link to where data requests can be made. Please update your statement with the missing information.

Response.

The data availability statement was revised to include contact information.

Reviewer comments

Reviewer #1: The Author did a great job with the methodology and analysis however the discussion needs more work. The discussion lacks a flow that engages the reader and does not enable ease in connecting the dots of the findings.

Response

Thank you for your comment. The Discussion section was revised to ensure proper flow and connections between different findings.

Reviewer #2: Kindly revise your article thoroughly following the pattern recommended by the PLOS ONE.

Response

The manuscript was revised to meet PLOS ONE's style and pattern requirements.

Sincerely yours,

Dr. Rami Saadeh
