# Supplementary material for: Knowledge and attitudes of medical students towards the health impact of climate change: A study from Jordan
Source: PLoS One. 2025 May 28;20(5):e0324943. doi: 10.1371/journal.pone.0324943 (PMC12118995; doi:10.1371/journal.pone.0324943)
Supplement: Attachment — Submitted filename: Responses_to_reviewers_auresp_2.docx [file pone.0324943.s004.docx]

**Dear Editor of PLOS-ONE**

We are thankful to the reviewers for their thoughtful and detailed review of our manuscript (ONE-D-24-44537 R1) entitled “Knowledge and attitudes of medical students towards the health impact of climate change: a study from Jordan”. All the recommended changes and edits have been incorporated in the revised version of our manuscript. We greatly appreciate the time and effort invested to produce this detailed review. Our point-by-point response is provided below.

Academic Editor
PLOS ONE

Comment

Journal Requirements:
Please review your reference list to ensure that it is complete and correct. If you have cited papers that have been retracted, please include the rationale for doing so in the manuscript text, or remove these references and replace them with relevant current references. Any changes to the reference list should be mentioned in the rebuttal letter that accompanies your revised manuscript. If you need to cite a retracted article, indicate the article’s retracted status in the References list and also include a citation and full reference for the retraction notice.

Response

Thank you. References were checked. All references are now complete and correct. We don’t have retracted papers among the references.


Reviewers' comments:
Reviewer #2:

Comment

1.Kindly add continuous line numbers to the manuscript

Response

Thank you. Continuous line numbers were added to the manuscript.

Comment
2.ABSTRACT: Kindly add about methodology.

Response

The Methodology part was expanded in the Abstract as suggested.

Comment

3.INTRODUCTION: Kindly Write a strong rationale

Response.

The rational in the Introduction was revised as suggested.

Comment

4.Study design is mentioned in caption but not written anywhere. Kindly add name of the study design used.

Response

The name of study design was added to the Method section

Comment
5.Conclusion may kindly be strengthened.

Response

The Conclusion section was revised as suggested.

Reviewer #3:

Comment

The manuscript addresses an important and timely topic, focusing on medical students’ awareness and attitudes toward the health impacts of climate change, particularly in the underrepresented MENA region. The methodology is robust, with a clearly defined study population, ethical approval, and validated data collection tools. The discussion ties the findings to relevant global and regional studies, emphasizing the need for integrating climate change into medical curricula.
However, the same suggestions for improvement is to address typographical and grammatical errors such as in introduction Sentence: "Phenomena associated with climate change are plenty and include extreme weather (heat waves, cold weaves, and floods)..."Issue: "Cold weaves" should be "cold waves."

Response

Thank you. The indicated sentence was revised as indicated

Comment

In methods, Sentence: "Before distributing the questionnaire, the instrument was reviewed by two experts in the field to verify its clarity and content. "Issue: No grammatical error, but "clarity and content" could be expanded for specificity. Suggestion: "Before distributing the questionnaire, the instrument was reviewed by two experts in the field to verify its clarity, relevance, and content accuracy."

Response

The indicated sentence was revised as suggested.

Comment

In results, Sentence: "The percentages of male and female students were comparable (47.1% and 52.8% respectively)."Issue: A comma is missing after "52.8%."

Response.

The sentence was revised as suggested.

Sincerely yours,

Dr. Rami Saadeh
