# Supplementary material for: Knowledge and attitudes of medical students towards the health impact of climate change: A study from Jordan
Source: PLoS One. 2025 May 28;20(5):e0324943. doi: 10.1371/journal.pone.0324943 (PMC12118995; doi:10.1371/journal.pone.0324943)
Supplement: Attachment — Submitted filename: Plos R3 responses.docx [file pone.0324943.s005.docx]

**Dear Editor of PLOS-ONE**

We are thankful to the reviewers for their thoughtful and detailed review of our manuscript (ONE-D-24-44537 R2) entitled “Knowledge and attitudes of medical students towards the health impact of climate change: a study from Jordan”. All the recommended changes and edits have been incorporated in the revised version of our manuscript. We greatly appreciate the time and effort invested to produce this detailed review. Our point-by-point response is provided below.

**Journal Requirements:**

Please review your reference list to ensure that it is complete and correct. If you have cited papers that have been retracted, please include the rationale for doing so in the manuscript text, or remove these references and replace them with relevant current references. Any changes to the reference list should be mentioned in the rebuttal letter that accompanies your revised manuscript. If you need to cite a retracted article, indicate the article’s retracted status in the References list and also include a citation and full reference for the retraction notice.

**Response**

The references were checked and formatted according to PLOS style. They are complete and correct. None of the cited papers have been retracted.

**Reviewers' comments:**

Reviewer #2: Dear Author, language may kindly be further improved and refined.
xxxxxxxxxxxxxThanks xxxxxxxxxxxxxx

Reviewer #4: The study addresses an important public health issue and fills a gap in the MENA region regarding the awareness of climate change impacts on health among medical students.
There are typographical and grammatical issues throughout, such as “cold weaves” instead of “cold waves” and inconsistent use of singular/plural nouns. I suggest proofreading 'optional'

Response

The manuscript was checked for English. Changes were highlighted in the revised manuscript.

Sincerely yours,

Corresponding author
